# Application of venovenous extracorporeal membrane oxygenation combined with continuous renal replacement therapy in a high-risk liver transplant recipient: a case report

**DOI:** 10.3389/frtra.2025.1683395

**Published:** 2025-10-10

**Authors:** Li Wen Yang, Yunli Zhang, Lijun Lu, Bin Xiong

**Affiliations:** People’s Hospital of Guangxi Zhuang Autonomous Region, Nanning, China

**Keywords:** ECMO, liver transplantation, acute respiratory distress syndrome, CRRT, case report

## Abstract

**Introduction:**

This report describes the novel perioperative application of VV-ECMO combined with CRRT in a high-risk liver transplant recipient with irreversible hypoxemia and multi-organ dysfunction, expanding therapeutic options for traditionally contraindicated patients.

**Case presentation:**

A 27-year-old male with acute-on-chronic liver failure (chronic hepatitis B + alcoholic liver disease), hepatic encephalopathy, severe pulmonary infection, and coagulopathy developed life-threatening hypoxemia (PaO_2_ 60 mmHg on FiO_2_ 100%) during transplantation.

**Interventions:**

Emergency intraoperative VV-ECMO and postoperative CRRT were initiated.

**Outcomes:**

ECMO was withdrawn on postoperative day 4, the ventilator on day 11, and the patient was discharged on day 61. Follow-up showed normal liver function.

**Conclusion:**

Combined VV-ECMO/CRRT provides synergistic cardiopulmonary-renal support for high-risk liver transplants, creating a critical window for graft recovery. Multidisciplinary coordination is essential for success.

## 1 Introduction

Liver transplantation (LT) remains the sole curative treatment for end-stage liver disease (ESLD) (1). However, severe complications like acute respiratory distress syndrome (ARDS) or multi-organ dysfunction (MODS) often contraindicate LT (2). Extracorporeal membrane oxygenation (ECMO), particularly veno-venous (VV) mode, supports severe respiratory failure, while continuous renal replacement therapy (CRRT) manages fluid/electrolyte imbalances (3). A recent systematic review highlighted the growing use of ECMO as a rescue therapy in adult LT recipients, particularly in those with severe cardiopulmonary compromise, though perioperative application remains limited and poorly standardized (4). Although combined VV-ECMO/CRRT is established in cardiac surgery, its perioperative use in high-risk LT is scarcely reported (5). We present a case demonstrating its efficacy in salvaging a transplant recipient with refractory hypoxemia and metabolic crisis.

## 2 Patient information

### 2.1 Clinical course

A 27-year-old male with untreated chronic hepatitis B and alcoholic liver disease presented to an outside hospital on 2023-05-07 with 6 days of progressive jaundice, blurred vision, and left lower abdominal pain. Initial diagnoses included chronic viral hepatitis B, alcoholic optic neuropathy, alcoholic liver disease, and acute liver failure. After 48 h of conservative management without improvement, he developed impaired consciousness and agitation, prompting emergency transfer to our ICU on 2023-05-09. Preoperatively, the patient had moderate ARDS with a PaO_2_/FiO_2_ ratio of 200 mmHg. Although this represented significant hypoxemia, it was deemed temporarily acceptable for proceeding with surgery, and protective ventilation with fluid restriction was maintained. Given the considerable complexities and risks associated with intraoperative ECMO management during liver transplantation—such as anticoagulation-related bleeding and technical interference—the decision was made to proceed without preoperative ECMO bridge therapy.

On admission, physical examination revealed sedation (RASS-4), generalized icterus, bilateral pupils of 5.0 mm with sluggish light reflex, and coarse breath sounds bilaterally. The patient presented with Grade III–IV hepatic encephalopathy (West Haven Criteria), confirmed by elevated ammonia (206.5 µmol/L) and EEG showing diffuse slowing. Management included lactulose and rifaximin. Due to intracranial hypertension concerns, permissive hypercapnia and aggressive recruitment maneuvers were avoided to prevent exacerbating cerebral edema. Laboratory studies confirmed hyperbilirubinemia (TBIL 306.5 μmol/L, DIBL 165 μmol/L), elevated ammonia (206.5 μmol/L), leukocytosis (WBC 16.59 × 10⁹/L), coagulopathy (PT 26.3 s, APTT 43 s, FIB 1.88 g/L), and acute kidney injury (Cr 157 μmol/L). Chest/abdominal CT demonstrated bilateral pulmonary inflammation, colonic dilation, and chronic liver changes.

The patient was diagnosed with acute-on-chronic liver failure (ACLF) with hepatic encephalopathy, severe ARDS (PaO_2_/FiO_2_ 60 mmHg), AKI stage 2, and Staphylococcus aureus pulmonary infection (sputum Gram stain positive), with differential considerations including fulminant viral hepatitis and sepsis-induced multi-organ dysfunction. On 2023-05-11, he underwent allogeneic orthotopic liver transplantation under general anesthesia (operative time: 647 min; anhepatic phase: 33 min).

### 2.2 Intraoperative course and ECMO initiation

During the anhepatic phase, the patient developed progressive hypoxemia (PaO_2_ 102 mmHg on FiO_2_ 100%, PEEP 12 cmH_2_O), which further deteriorated following reperfusion (PaO_2_ 54 mmHg, pH 7.29, lactate 12.8 mmol/L), indicating impending cardiorespiratory collapse. Despite aggressive intraoperative interventions that were feasible in the surgical setting, such as high PEEP and neuromuscular blockade, oxygenation continued to decline. Conventional rescue therapies like prone positioning were not feasible during surgery. Immediately after surgery, multidisciplinary consensus was reached for emergent VV-ECMO initiation to prevent irreversible hypoxic injury and provide a bridge to graft recovery. A pre-existing triple-lumen central venous catheter was present in the right IJ. This was removed under ultrasound guidance, and the ECMO return cannula was inserted via a new puncture site cephalad to the previous insertion point to minimize infection risk. Cannulation involved ultrasound-guided right femoral vein drainage (21Fr multi-stage cannula advanced 35 cm to infrarenal IVC) and right internal jugular vein return (17Fr cannula, depth 14 cm), using a Bioline-coated circuit with Maquet Rotaflow® centrifugal pump (3,000–3,500 RPM) and Maquet Quadrox® polymethylpentene oxygenator (blood flow: 4.8–5.0 L/min) which resulted in immediate improvement in oxygenation (PaO_2_ 199 mmHg).

### 2.3 Postoperative management and CRRT integration

Continuous renal replacement therapy (CRRT) was initiated postoperatively using the Prismaflex® system (Baxter) in continuous venovenous hemodiafiltration (CVVHDF) mode. The CRRT circuit was integrated into the ECMO circuit using a sterile bridge tubing system, with the blood extraction line connected after the oxygenator and the return line connected before the oxygenator but after the ECMO pump, thereby avoiding additional vascular access. This configuration is recommended to ensure safety and effectiveness when combining the two circuits (6). The CRRT was performed using an Ultraflux AV600S filter (Fresenius Medical Care) in CVVHDF mode and with a cumulative ultrafiltration volume of 6,205 ml over the course of therapy (Table 1). Heparin infusion was initiated at 10 U/kg/h and titrated to maintain ACT between 160 and 180 s. Pre-ECMO coagulation profile showed PT 26.3 s, APTT 43 s, FIB 1.88 g/L. Post-ECMO, APTT ranged 45–60 s. No clinical bleeding or thrombosis occurred. Transfusion requirements included 2 units of FFP and 1 unit of platelets post-cannulation. Anticoagulation was managed using ACT-targeted heparinization (maintained between 160 and 180 s), with close monitoring of platelet (maintained >50 × 10⁹/L) and fibrinogen levels (maintained >1.5 g/L) to balance thrombotic and bleeding risks.

**Table 1:** Daily fluid balance and CRRT parameters.

Date	Total intake (ml)	Urine output (ml)	Ultrafiltration (ml)^a^	Total output (ml)	Fluid balance (ml)
Pre-operative	1,904	3,125	375	3,500	−1,596
Postoperative day 1	3,929	2,950	3,970	7,020	−3,091
Postoperative day 2	2,103	3,300	541	4,141	−2,038
Postoperative day 3	5,452.5	3,125	1,369	6,174	−721.5
Postoperative day 4	4,158.2	3,130	50	3,890	+268.2
Postoperative day 5	2,858	2,070	0 (CRRT stopped)	3,010	−152
Postoperative day 6	3,157	4,050	0	4,240	−1,083
Postoperative day 7	3,602	4,000	0	4,800	−1,198
Postoperative day 8	3,115	2,815	0	3,415	−300

^a^ Ultrafiltration volume reflects fluid removal by CRRT.

Pre-CRRT, the patient exhibited fluid overload (cumulative balance +2.5 L), metabolic acidosis (pH 7.25, BE −6.6 mmol/L), and worsening creatinine (peak 97 µmol/L). CRRT was initiated with CVVHDF, resulting in improved acid-base balance (pH 7.41, BE +0.6 mmol/L) and volume status (net negative balance by POD 3).

Cannula positioning was confirmed by transabdominal Doppler and x-ray, achieving immediate oxygenation improvement (PaO_2_ ↑199 mmHg) and enabling protective ventilation (FiO_2_ 40%, PEEP 8 cmH_2_O). Postoperatively, CRRT was initiated for fluid overload and metabolic acidosis (CVVHDF, blood flow 150 ml/min). alongside pharmacotherapy with vancomycin (1 g q12 h), caspofungin (70 mg LD/50 mg MD), tacrolimus (1 mg BD), and N-acetylcysteine infusion.

**Figure 1:**
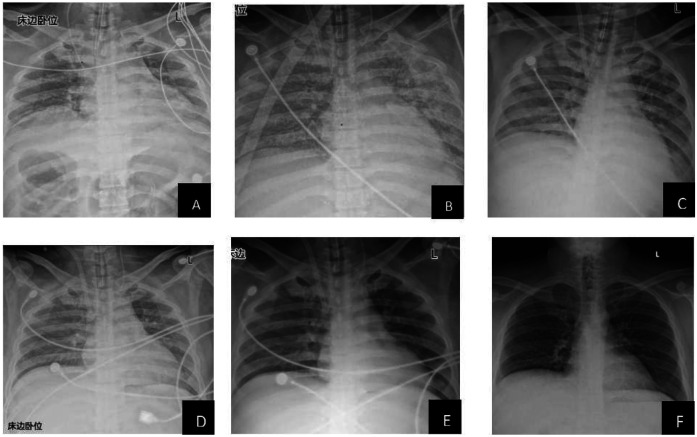
Radiological changes in the patient's lungs (**A**: preoperative; **B**: 1 day postoperatively; **C**: 2 days postoperatively; **D**: 3 days postoperatively; **E**: 4 days postoperatively; **F**: 9 days postoperatively). This study was approved by the relevant institutional review board/ethics committee, and informed consent was obtained from the patient.

The patient's lung condition improved rapidly (as shown in Figure 1). ECMO was successfully weaned on postoperative day (POD) 4 (PaO_2_ 129 mmHg on FiO_2_ 40%), followed by extubation on POD 11 with intact neurological status. The patient was transferred from the ICU on POD 14 and discharged on POD 61 with normalized liver function (TBIL 22.2 μmol/L, ALT 214 U/L) and no infection recurrence. Three-month follow-up confirmed asymptomatic recovery, graft viability on ultrasound, and normalized AFP (12 μg/L). Key laboratory trends included bilirubin decline (peak 312.2 μmol/L pre-op → 70.6 μmol/L POD 7), renal recovery (Cr 157 → 64 μmol/L), and coagulopathy resolution (PT 26.3 → 16.8 s POD 5) (Table 2).

**Table 2:** Postoperative trends in renal, hepatic, and coagulation parameters.

Parameter	Unit	Reference Range	Postoperative day 1	Postoperative day 2	Postoperative day 3	Postoperative day 4	Postoperative day 5	Postoperative day 6	Postoperative day 7
Renal function									
Creatinine (Cr)	μmol/L	59–104	48	97	66	54	56	64	82
BUN	mmol/L	2.5–7.1	3.7	6.6	9.0	10.4	10.9	10.1	9.4
Liver function									
Total Bilirubin (TBIL)	μmol/L	5–21	218.6	80.4	54.9	36.8	42.5	60.2	70.6
AST	U/L	15–40	44	249	290	254	128	110	215
ALT	U/L	9–50	92	629	204	239	204	182	214
Coagulation									
PT	s	11–14.5	22.5	17.1	16.0	16.9	16.8	18.0	21.5
APTT	s	29–42	>180	47.4	46.3	50.7	57.3	44.7	44.3
Fibrinogen (FIB)	g/L	2.0–4.0	1.71	2.04	1.86	1.61	2.13	3.58	4.41

## 3 Discussion

The successful integration of venovenous extracorporeal membrane oxygenation (VV-ECMO) and continuous renal replacement therapy (CRRT) in this high-risk liver transplant (LT) recipient exemplifies a paradigm shift in managing multisystem organ failure in end-stage liver disease (ESLD), underscoring the potential for bridging critically ill candidates through perioperative crises but also highlighting the imperative for meticulous patient selection and techno-physiological optimization.

The successful outcome in this critically ill patient, contrasting sharply with the high mortality [e.g., 63% in pediatric LT-ECMO recipients per ELSO Registry (7)], can be attributed to the synergistic physiological effects of combined ECMO/CRRT support. Three key factors likely contributed to this divergence:

1. Proactive ECMO Initiation: Despite attempts with conventional rescue therapies, the patient's hypoxemia progressed rapidly. Proactive deployment immediately post-surgery during the reperfusion crisis (vs. delayed postoperative rescue) aligns with evidence where early ECMO for reversible respiratory failure yielded significantly higher survival (68% vs. 32%) (8), preempting cumulative organ injury; Furthermore, while not directly comparable due to differences in study design and population, the outcome in our case appears favorable when considered alongside the findings of a recent systematic review by Reid et al. (4), which reported an overall 90-day mortality of 45.9% in adult LT recipients on ECMO. This difference might be partly explained by the predominantly postoperative ECMO initiation in the review cohort, where patients often had established multiorgan failure, the strongest predictor of mortality. Our proactive intraoperative application before the full establishment of irreversible MSOF likely provided a critical advantage.
2. Integrated CRRT: Optimized fluid management prevented volume-related cardiac strain, addressing a major cause of ECMO-LT failure linked to renal/metabolic derangements. The integration of CRRT with ECMO is crucial in this population, as supported by a recent meta-analysis which demonstrated that concomitant CRRT was associated with significantly improved survival in patients receiving ECMO support (9). This finding is supported by a systematic review and meta-analysis by Liu et al. (2025) (10), which also reported an association between concurrent CRRT with ECMO with improved survival and fluid balance. In our case, this synergy was realized by integrating the CRRT circuit into the ECMO circuit, obviating the need for additional vascular access. This strategy effectively addressed the characteristic fluid overload and inflammatory milieu of ACLF, thereby mitigating a key pathway to MSOF and creating a more stable bridge to graft recovery.
3. Anticoagulation Precision: ACT-targeted heparinization (160–180 s) and maintenance of platelet (>50 × 10⁹/L) and fibrinogen (>1.5 g/L) levels, complemented by heparin-coated circuits (e.g., Bioline®), minimized surgical bleeding and thrombotic risks.

The combined use of ECMO and CRRT carries risks of circuit clotting, infection, and hemodynamic instability. In our patient, we mitigated these through strict anticoagulation monitoring, aseptic circuit management, and avoidance of additional vascular access by integrating CRRT into the ECMO circuit. No circuit-related complications occurred, underscoring the importance of meticulous multidisciplinary management.

Pathophysiologically, in acute-on-chronic liver failure (ACLF), the triad of hepatic encephalopathy, ARDS, and acute kidney injury perpetuates a vicious cycle of hypoxemia, cytokine storm, and fluid overload. In this case, VV-ECMO rapidly restored oxygenation (PaO_2_: 60 → 199 mmHg; Table 3), thereby mitigating graft ischemia-reperfusion injury and enabling protective ventilation to reduce ventilator-induced lung injury (VILI).

**Table 3:** Serial blood gas analysis and ventilator parameters.

Time point	pH	PaO_2_ (mmHg)	PaCO_2_ (mmHg)	BE (mmol/L)	Lactate (mmol/L)	OI^a^ (mmHg)	Ventilator mode	FiO_2_ (%)	PEEP (cmH_2_O)	VT (ml)
Preoperative	7.46	80	40	4.2	2.4	200	A/C	40	8	450
Post-incision	7.41	80	43	4.7	2.8	195.1	A/C	41	10	420
Anhepatic phase	7.24	102	41	−9.6	8.3	102	A/C	100	12	400
Post-reperfusion	7.29	54	50	−2.9	12.8	50	A/C	100	14	380
End of surgery	7.27	77	34	7.0	14.8	77	A/C	100	14	380
Post-ECMO initiation	7.49	199	55	18.6	12.3	199	A/C	40	8	300
Postoperative day 1	7.64	88	35	17.4	3.0	88	SIMV	40	8	300
Postoperative day 2	7.25	115	47	−6.6	0.8	115	SIMV	40	8	300
Postoperative day 4 (pre-weaning)	7.41	136	41	0.6	0.9	272	SIMV	40	8	400
Post-ECMO withdrawal	7.40	129	48	5.3	30.3	323	SIMV	40	8	400

^a^ OI, oxygenation index (PaO_2_/FiO_2_).

The decision to initiate ECMO intraoperatively, though outside conventional ELSO timing guidelines, was driven by the acute reversibility of the respiratory failure and the imminent risk of reperfusion-induced cardiac arrest. This proactive deployment during the reperfusion crisis aligns with evidence that early ECMO for reversible respiratory failure improves survival by preempting cumulative organ injury.

Furthermore, our experience suggests that the integrated ECMO-CRRT support system creates a crucial bridge to graft recovery, which is aligned with emerging literature (11). This approach may expand the boundaries of transplant eligibility for critically ill patients who would otherwise be deemed inoperable, particularly those requiring extended-criteria grafts or combined organ transplantation.

Concurrently, CRRT addressed the critical “third-hit” phenomenon in LT by modulating fluid balance (cumulative ultrafiltration: 6,205 ml) (Table 1), correcting acid-base derangements, and clearing ammonia and inflammatory mediators (e.g., IL-6, TNF-α), thereby attenuating systemic inflammation and supporting hepatic regeneration.

This synergistic support created a physiological window for graft recovery, evidenced by declining bilirubin (312.2 → 70.6 μmol/L) and normalized coagulation (PT: 26.3 → 16.8 s).

This case broadens ECMO applicability, supporting the use of extended-criteria grafts (e.g., high-MELD, steatotic livers) and combined organ transplants (e.g., liver-lung) (12), and potentially mitigating hypoxemic neuronal injury in peri-arrest scenarios, contrasting with the poor outcomes (100% mortality) of ECPR post-LT (13). Persistent barriers include significant cost-utility concerns (>$10,000/day), necessitating protocols for rapid weaning (as achieved here within 4 days), the need for validated biomarkers (e.g., intraoperative lactate >12.8 mmol/L predicting crisis) to guide ECMO timing, and the requirement for better technological integration, potentially via hybrid ECMO-CRRT devices currently under investigation (e.g., NCT04823017) (14).

## 4 Conclusion

VV-ECMO coupled with CRRT represents a paradigm shift in managing high-risk LT recipients with multisystem failure. Its success hinges on: (1) preemptive deployment for reversible insults, (2) precision management of anticoagulation/fluid balance, and (3) multidisciplinary readiness. While resource-intensive, this approach may expand LT access to critically ill patients previously deemed ineligible. Prospective studies comparing ECMO strategies in LT are urgently needed.

## 5 Patient perspective

The patient expressed profound gratitude for the life-saving intervention and detailed understanding of the complex treatment.

## Data Availability

The original contributions presented in the study are included in the article/Supplementary Material, further inquiries can be directed to the corresponding author.
